# Multi-scalar Analysis of a Southern African Assemblage: The Middle Stone Age at Ha Makotoko, Lesotho

**DOI:** 10.1007/s41982-025-00238-x

**Published:** 2025-11-20

**Authors:** Tjaark Siemssen, Charles Arthur, Genevieve Dewar, Marlize Lombard, Peter Mitchell

**Affiliations:** 1 School of Archaeology, University of Oxford, Oxford, UK; 2 Institute for Prehistory, University of Cologne, Cologne, Germany; 3 Cultural Heritage Department (Europe, Middle East and Africa), Environmental Resources Management, Cardiff, UK; 4 Rock Art Research Institute, School of Geography, Archaeology, and Environmental Studies, University of the Witwatersrand, Johannesburg, South Africa; 5 Department of Anthropology, University of Toronto Scarborough, Toronto, Canada; 6 Palaeo-Research Institute, University of Johannesburg, Johannesburg, South Africa

**Keywords:** Lithic analysis, Middle Stone Age, Post-Howiesons Poort, Southern Africa, Marine Isotope Stage 3, Lesotho

## Abstract

This study describes a Middle Stone Age (MSA) assemblage from Ha Makotoko, Lesotho. Excavated in 2010, the assemblage dates to > 42 ka and falls within Marine Isotope Stage 3. Faunal remains indicate a grassland environment during the time of occupation. The lithic artefacts found at Ha Makotoko comprise a predominantly flake-based industry with some blades and an increasing frequency of bladelets throughout the sequence. Artefacts were primarily made on fine-grained materials and cores were mainly struck irregularly. The few tools present include points, and miscellaneously retouched pieces are common. Use-wear traces on points show direct evidence of hafting, with butchering and woodworking indicated. The MSA assemblage from Ha Makotoko is compared to material from the nearby sites of Ntloana Tšoana and Rose Cottage Cave, as well as sites further to the east in the Maloti-Drakensberg Mountains and along South Africa’s southeastern coast. These comparisons confirm its association with the post-Howiesons Poort technocomplex of Marine Isotope Stage 3 and reveal similarities to the final MSA. However, the high amount of informal retouch and other differences further complexify what is already considered a technologically highly variable period. Presenting this assemblage here adds additional evidence for a phase of the Middle Stone Age that remains poorly understood in its entirety. Situated along the intersection of Afroalpine and grassland environments in western Lesotho, Ha Makotoko offers a bridging record for these contrasting ecologies that highlights the diverse applicability of post-Howiesons Poort and final MSA toolkits.

**Supplementary Information:**

The online version contains supplementary material available at 10.1007/s41982-025-00238-x.

## Introduction

The Middle Stone Age (MSA) of southern Africa marks the emergence of *Homo sapiens* and sees the transition from bifaces to flake- and blade-based lithic industries (Lombard et al., 2022; Mitchell, 2024). Accordingly, various sub-periods of the MSA have received considerable archaeological attention. The Still Bay and Howiesons Poort technocomplexes have, for example, frequently been named as hallmarks of the onset of a now largely discredited suite of ‘modern behaviours’ (McBrearty & Brooks, 2000; Henshilwood & Marean, 2003; Mellars, 2006; Wurz, 2008; Porraz et al., 2013; but see Shea, 2011; Wadley, 2013; Garofoli, 2016; Roberts, 2016). Focusing attention on such assemblages which broadly date to Marine Isotope Stages (MIS) 5a (c. 88–71 ka) and 4 (c. 71–57 ka) respectively has, however, left later components of the Middle Stone Age poorly understood, even though the post-Howiesons Poort phenomenon of MIS 3 (c. 57–29 ka) presents archaeologists with a degree of technological diversification that stands in stark contrast to the greater uniformity of the preceding Howiesons Poort (Lombard & Parsons, 2011; Mitchell, 2008).

**Fig. 1:**
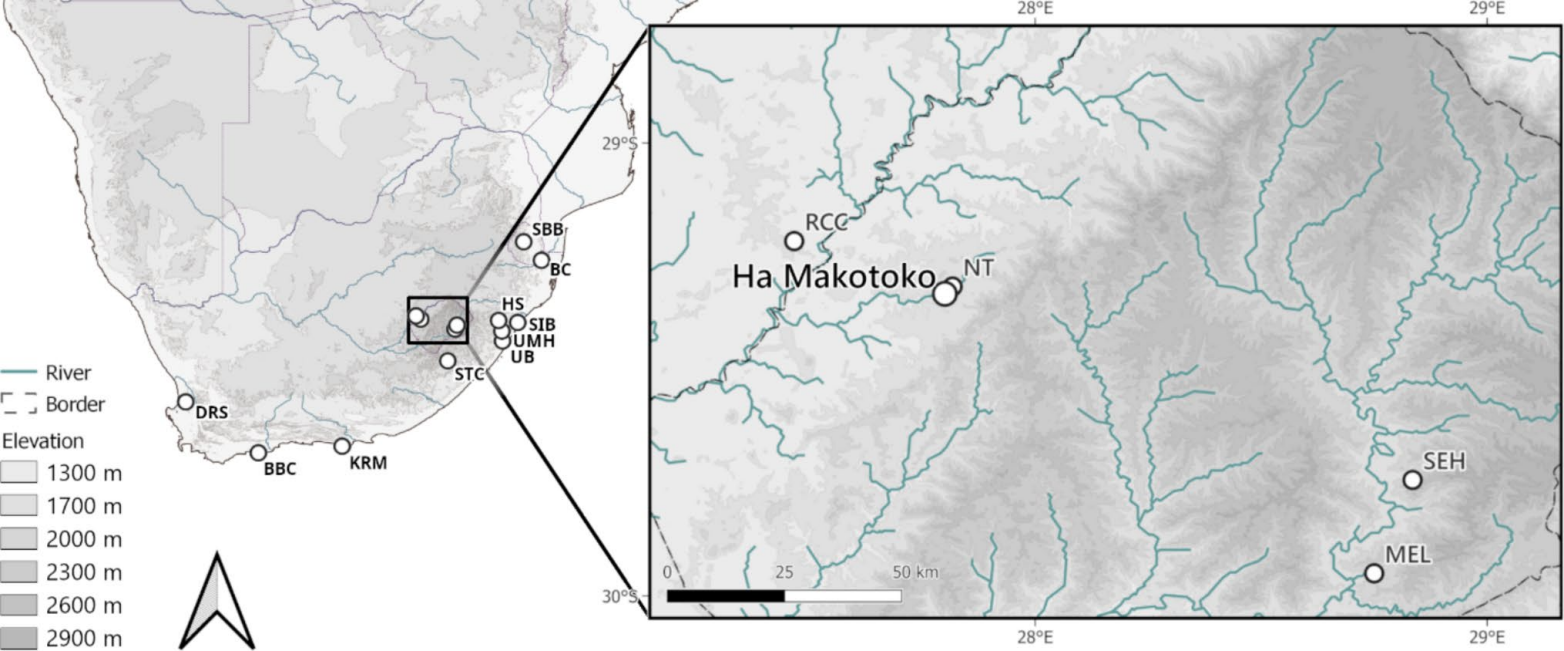
Southern Africa, with the research area highlighted on the right, showing the location of Ha Makotoko. Other site names mentioned in this text are abbreviated: BBC, Blombos Cave; BC, Border Cave; DRS, Diepkloof Rock Shelter; HS, Holley Shelter; KRM, Klasies River Mouth; MEL, Melikane; NT, Ntloana Tšoana; RCC, Rose Cottage Cave; SEH, Sehonghong; SBB, Sibebe; SIB, Sibhudu; STC, Strathalan Cave; UB, Umbeli Belli; UMH, Umhlatuzana

Since 2010, interest in the period immediately following the Howiesons Poort has increased substantially. Across southernmost Africa, sites such as Rose Cottage Cave, Border Cave, Sibhudu, Klasies River Mouth, and Diepkloof have contributed significantly to our understanding of the post-Howiesons Poort of earlier MIS 3 (Fig. 1). Despite regional specificities, the post-Howiesons Poort is characterised by multiple core reduction schemes including Levallois production, discoid, and platform cores, with a focus on unifacially retouched, mostly elongated points (Soriano et al., 2007; Villa et al., 2010; Conard et al., 2012; Porraz et al., 2013; de la Peña et al., 2022; Lombard et al., 2022). Around 40 ka, further technological shifts observed at sites like Border Cave and Melikane see another proliferation of microlithic technology, which is largely uncommon during the post-Howiesons Poort but considered by some to be an early expression of the Later Stone Age (cf. Bousman & Brink, 2018; Villa et al., 2012). Others, in turn, posit a more gradual or much later onset of the MSA/LSA transition (Blessing et al., 2023; Pazan et al., 2023). Instead of identifying an early LSA expression, they characterise the period from around 40 ka as the *late* or *final* MSA, characterised by production of both unifacial and bifacial points, increasing relevance of bipolar knapping, and increasing frequency of bladelets in assemblages. Yet, this period in particular is known for its high variability across sites (Bader et al., 2022a, 2024; Lombard et al., 2022; Villa et al., 2005; Wadley, 2005), and, despite numerous efforts, the post-Howiesons Poort, the late MSA, and the final MSA are all similarly defined, with inconsistent use of definitions persisting in the literature owing partly to the increasing variability of stone tool technologies across MIS 3 (Lombard et al., 2022; Will et al., 2024).

This is also due to a lack of detailed regional chronologies and, whilst sites from the eastern part of South Africa have undoubtedly helped to improve the resolution of current understandings, western Lesotho in particular provides a bridging record from a location that lies in a contact zone between the final MSA and microlithic expressions at Melikane and Border Cave. The site presented in this study describes an early- to mid-MIS 3 lithic assemblage excavated in 2010 at Ha Makotoko, Lesotho, that contains at least two occupational phases. Here, our concern is specifically with the technological relationship between those two phases. Further, building on the burgeoning record of MIS 3 sites published in recent years, the record from Ha Makotoko is then compared to sites in both the local context of the wider Caledon Valley as well as more broadly across southeastern southern Africa. Guiding this investigation is the question of how far assemblages associated with the post-Howiesons Poort, the late MSA, and the final MSA are comparable on a regional and temporal scale. Our study presents additional evidence for a period that remains vaguely understood to this day, in an ecologically sensitive location at the intersection of Afroalpine and grassland environments, a location that was subject to considerable environmental fluctuations during MIS 3 (Stewart & Mitchell, 2018).

## The Site of Ha Makotoko

**Fig. 2:**
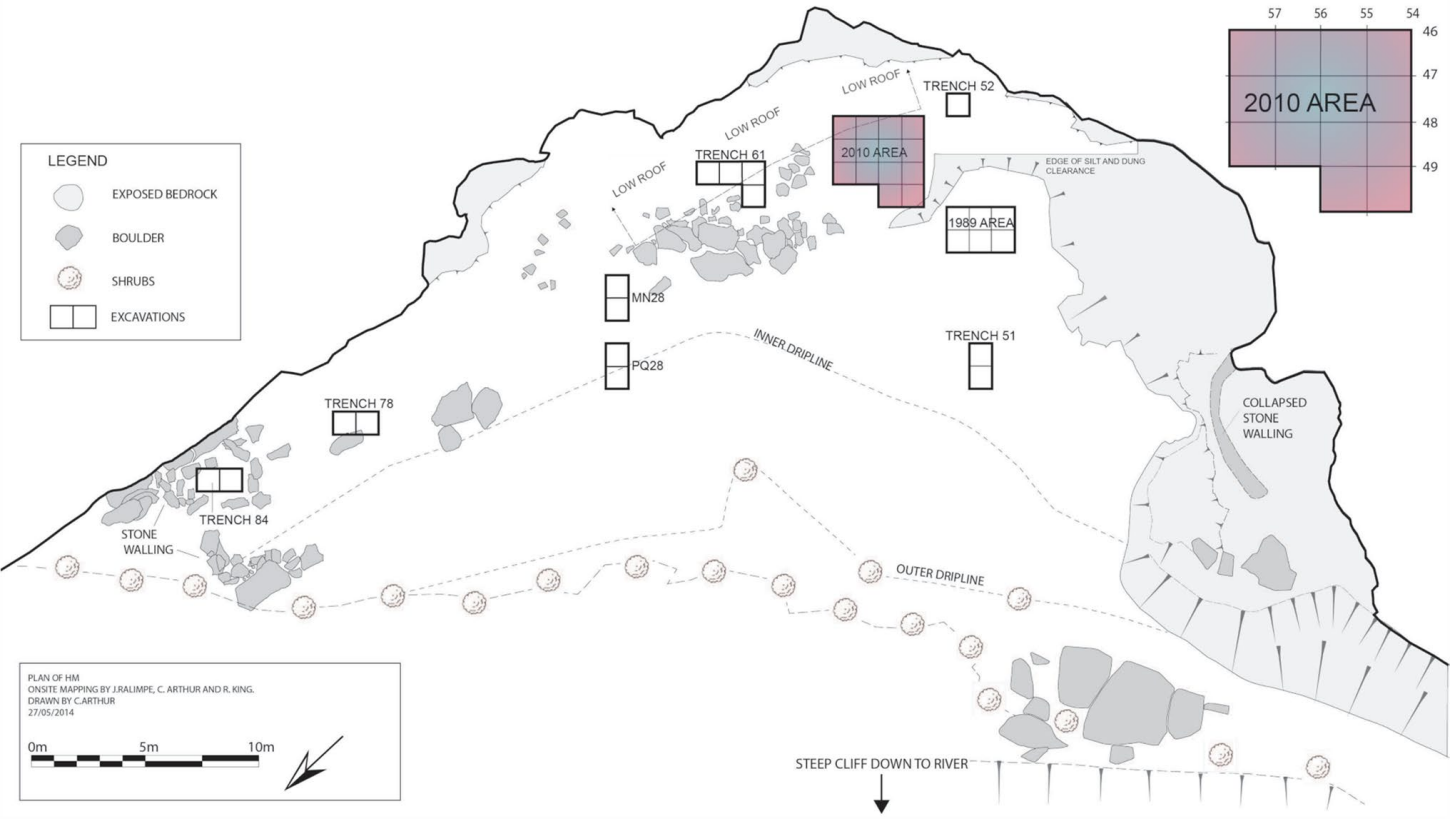
Plan of Ha Makotoko, with the area excavated in 2010 highlighted. Figure by Charles Arthur

Ha Makotoko was first excavated in 1989, but those excavations only encountered in situ archaeological deposits of early Holocene age, with just a tiny number of MSA artefacts at their base, assumed at the time to have been introduced to the site from the surrounding landscape where several open-air MSA scatters exist (Mitchell, 1993). Much more extensive excavations followed in 2010 prior to the construction of the Metolong Dam and the impoundment of its associated reservoir, which now covers a 14-km-long stretch of the Phuthiatsana Valley and has submerged both Ha Makotoko and the nearby site of Ntloana Tšoana, among other rock-shelters (Arthur, 2018, 2022; Arthur et al., 2011, 2018; Mitchell & Arthur, 2014). Ha Makotoko was tucked into a bend of the Phuthiatsana River, a tributary of the Caledon, which forms Lesotho’s western border with South Africa, at an elevation of about 1640 m above mean sea level (a.s.l.). Located approximately 10 km west of the Front Range of the Maloti Mountains (Fig. 1), the rock shelter comprised an area of some 820 m^2^ and faced the valley with a north-west orientation (Fig. 2). The site itself, as well as the surrounding area, lay in the sandstones of the Clarens Formation, which belongs to the wider Karoo supergroup (Mitchell & Arthur, 2014). Today, the Phuthiatsana Valley experiences up to around 700 mm of rain annually, with most of it falling in summer. Mean annual temperatures range around 17 °C, with June being the coldest month (9 °C mean) and January the warmest (23 °C mean; Dewar et al., 2024; Patalano et al., 2023). The plateau above the Phuthiatsana River, as well as small areas along it, are today intensively farmed for maize and other crops, having been settled by Sotho-speaking farmers in the first half of the nineteenth century (Gill & Nthoana, 2010; Maggs, 1976). As of the Late Holocene, local vegetation largely comprises C_4_ plants such as grasses with a high water-use efficiency (Patalano et al., 2023).

Due to the construction of the Metolong Dam, the results discussed here form part of a final chance to investigate this stretch of the Phuthiatsana River. Ha Makotoko, with its 60-m-wide dripline, was the largest rock shelter along the river. Its re-excavation showed that its stratigraphy extended from around the start of MIS 3 to the Holocene, with more recent hunter-gatherer fine-line rock paintings executed on its walls during the last ~ 1000 years (Bonneau et al., 2017).

### Stratigraphy

**Fig. 3:**
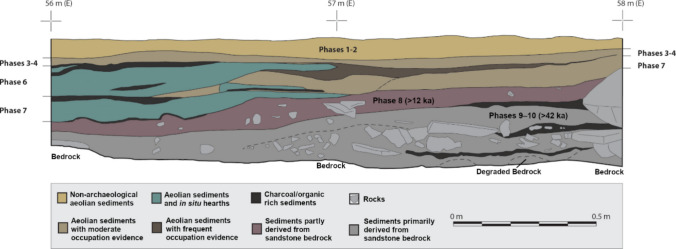
Ha Makotoko: stratigraphy of the 2010 excavation area. Phases 9 and 10 correspond to the post-Howiesons Poort. After: Mitchell and Arthur (2014)

As previously indicated, renewed excavation of Ha Makotoko unexpectedly revealed the presence of intact Pleistocene deposits in parts of the site not sampled in 1989. Figure 2 shows the locations of these excavation areas. The material discussed here comes from the 2010 area highlighted in the map and involves Squares 55/49, 54/49, and 57/48 to 54/46 (also referred to as Area 54). Excavation took place in 1 m^2^ squares comprising four quadrants each measuring 50 by 50 cm. Contexts were differentiated by sediment colour and texture where feasible, and sub-divided into successive arbitrary spits of 25 mm thickness when larger than this. All sediment was dry sieved on site using a 2-mm mesh, with finds subsequently temporarily exported from Lesotho for analysis. Following excavation, recovered contexts were grouped into ten Phases on the basis of overall sedimentological characteristics and artefact content (Fig. 3). The material discussed here comes from deposits assigned to Phases 9 and 10.

Phase 9 comprises Contexts 83, 65, 60, 54, 173, 172, 192, 205, 195, 200, 201, 196, 52, 84, 85, 190, and 209 (from youngest to oldest) and its sediment contains some weathered material from the shelter’s roof and floor. There are two hearths, Context 60 and Context 65, both of which were likely only briefly used. Nearby, Context 83 comprised loose grey and red fine sand with a high concentration of uncarbonised plant material that appears to have been the remains of grass bedding. Slightly lower down the sequence, but below an intervening sandstone-rich moderately to loosely compacted sand that recalled Context 56 in Phase 8 and was removed as Context 173, a series of contexts formed the fill of a large pit (Context 196). Large, but shallow, and oval in shape, this feature extended over at least 1.13 by 0.82 m, but reached beyond the main excavation area. Filled with grass, it seems likely to have been either used for storage or, perhaps more likely, as a sleeping hollow, in which case the grass presumably again reflects the presence of bedding material. Likely associated in time is Context 172, a localised area of yellowish brown to blackish grey fine sand rich in charcoal and bone interpreted as a refuse dump. Underlying all these contexts, Context 52 was a compact reddish brown gritty sand that contained a small silt and clay fraction as well as numerous small sandstone inclusions. It appears to have been largely of geological origin and clearly derived from weathering of the shelter’s bedrock. A small hearth below it in Square 57/48 represented the initial use of the main excavation area during this phase of the site’s history.

Phase 10, comprising the underlying Contexts 78, 128, 88, and 90, marked the first phase of building up deposits within the main excavation area. Its sediment was generally compact, but looser in places, and consisted of greyish-brown gritty but fine sand with frequent sandstone inclusions, largely geological in origin. Despite this, it showed a higher artefact frequency than in the sedimentologically similar Context 52 higher up. Phase 10 was poorly differentiated and was removed in spits of 25 mm thickness as a result; it seems likely that it built up over a considerable time. Excavation ceased at the base of the Phase 10 deposits on encountering bedrock.

The Phase 8 assemblage from Ha Makotoko contains layers marked by successive increases in bladelet frequency. Its top is dated to c. 12 ka cal BP, and with the dating sample OxA-27318 from context 190 (upper Phase 9) indicating an age of 43 ka cal BP, Phase 8 can only vaguely be associated with the MIS 3/MIS 2 transition, including the Last Glacial Maximum. The material was not used in this study, but was analysed separately by Seneši (2023) and Dewar et al. (2024) who identified it as a Robberg assemblage.

Three radiocarbon dates from the 2010 excavations at Ha Makotoko are available for the layers associated with the MIS 3 assemblages discussed here from the 2010 area, with one additional date from Trench 61, a few metres to the east (Table 1). Two are associated with Phase 9. Belonging to Context 60 (laboratory number UGAMS-8988) and Context 65 (laboratory number OxA-27317) respectively, they securely date Phase 9 to around 43 ka cal BP. A third radiocarbon date associated with the stratigraphically lower Context 190, just above the end of Phase 10, can only be said to be older than 52 ka (laboratory number OxA-27318) as it approaches the boundary of radiocarbon dating. This ambiguity limits the wider chronological inferences that we can make for Phase 10, and comparability between the two phases is therefore assessed through their technological analysis in this study.

**Table 1:** The four radiocarbon dates from Ha Makotoko associated with the post-Howiesons Poort. Dates have been calibrated with SHCal20 (Hogg et al., 2020)

Laboratory number	Phase	Context	Age BP	Calibrated age *p* = 95.4%	Sample material (all charcoal)
UGAMS-8988	9	060	40,100 ± 230	42,846–43,913 cal BP	Unidentified
OxA-27317	9	065	39,450 ± 700	42,310–44,150 cal BP	*Clutia pulchella*
OxA-27318	9	190	> 52,200	-	*Clutia pulchella*
UGAMS-11596	Trench 61	181	39,620 ± 210	42,646–43,120 cal BP	*Euphorbia* sp.

### Palaeoenvironmental Background

Investigation of plant wax *n*-alkanes and hydrogen isotopes from the sediments of Ha Makotoko by Patalano et al. (2023) provides insights into the C_3_ and C_4_ vegetation present in and around the site over time. These archaeobotanical remains indicate that the region was drier during the Pleistocene than during the Late Holocene but that it would have experienced consistent precipitation during MIS 3 (Patalano et al., 2023). Patalano et al. (2023) see this as an indicator of the reliability of local water sources and highlight how Ha Makotoko’s location between the higher mountain ranges of the Maloti-Drakensberg Massif and the lowlands of western Lesotho may have meant that this offered an advantageous biome diversity for hunter-gatherers during MIS 3, especially during repeated drier episodes.

The availability of this site-specific record from Ha Makotoko provides a rare opportunity to investigate the relationship between occupational history and environmental conditions, as past studies have shown significant temperature variation in different regions of southern Africa (e.g. Chevalier & Chase, 2015). It is complemented by the faunal record available from Ha Makotoko that is discussed in a later section of this study.

The two MIS 3 pulses at Ha Makotoko coincide with shifting ecological conditions in the region. A synthesis of relevant palaeoclimatic and palaeoenvironmental data by Stewart and Mitchell (2018) concluded that the Maloti-Drakensberg region as a whole is particularly susceptible to climate change due to its stark differences in altitude and topography. Such environmental contrasts may have led to unexpected human-environment feedbacks in this region. Marine Isotope Stage 3 appears to have been a quite volatile period prone to changes in both precipitation and temperature, with more humid conditions potentially prevailing early on followed by a cooler episode associated with widespread aridity from around 43 ka (Stewart & Mitchell, 2018). This is also evidenced by records from neighbouring locations, such as at ‘Site 1’ near Thabana Ntlenyana, Lesotho’s highest mountain (3482 m a.s.l.), where sediment records suggest a shift towards a drier, colder climate around 42 ka. Further south in the Senqu (Orange) Valley, the site of Melikane, too, shows changes in site formation processes around this time, further supporting the shift from a wetter and warmer climate towards more arid conditions, and fluctuating temperature, humidity, and atmospheric CO_2_ (Stewart et al., 2016). This shift is also reflected in the increased aeolian activity reported from the Golden Gate Highlands National Park in South Africa’s Free State province west of the Caledon River around this time (Stewart & Mitchell, 2018). Further records show that beyond the Maloti-Drakensberg Mountains, MIS 3 can generally be understood to be drier, and more unstable than the preceding MIS 4, and included a quasi-linear decrease in mean annual temperature of 2 °C from c. 45 to 20 ka, plus indications for a steady decrease of precipitation outside the Drakensberg Mountains (Chevalier & Chase, 2015). The Maloti-Drakensberg Mountains experience the coldest climate regime in southern Africa owing to their elevation.

### Lithic Assemblages

During the 2010 excavations at Ha Makotoko, 1648 individual lithic artefacts with a size of > 20 mm were recovered from Phases 9 and 10, each recorded in quarter square metres and ordered into numbered contexts. In the present study, these artefacts were analysed both metrically and in terms of their attributes (cf. Tafelmaier et al., 2022). Attributes recorded for each artefact were raw material type, cortex presence, cortex type, blank, tool type, weight, completeness (transverse/longitudinal), platform width, platform thickness, technological length, technological width, technological thickness, maximum dimension, maximum width, maximum thickness, core reduction type, core type, number of removal scars, number of striking platforms, initiation type, platform lip, bulb, termination, flake form, retouch angle, retouch position, retouch type, and edge damage (for a detailed key of attributes and variables, see the supplementary online material).

Most of these variables were selected as they have been shown to enable high replicability between analysts in a recent study on the analysis of unmodified flakes by Pargeter et al. (2023: 19). Definitions for core reduction types and core types have been taken from Shea (2013; see also Boëda, 1993). Retouch type and retouch angles also follow the classification by Shea (2013: 170). Raw material descriptions are adopted from earlier studies at Ha Makotoko (Mitchell, 1993; Mitchell & Arthur, 2014) and in the wider Maloti-Drakensberg research area (Mitchell, 1996; Villa et al., 2005; Wadley et al., 2017). Cortex classification follows Porraz et al. (2013).

The artefacts were recorded using the E5 software developed by McPherron (2022). The data were visualised using R-Studio (R Core Team, 2021) and QGIS (QGIS Development Team, 2023). Following the metric and attribute analysis, selected artefacts underwent visual documentation, which included photography and 3D modelling through structure from motion using the photogrammetry workflow for lithic artefacts by Porter et al. (2016). Visualisation of artefacts in three dimensions, particularly of cores, provides more information and is increasingly becoming a standard for digital inspection of lithic artefacts. 3D models generated for this study can be accessed via a Zenodo repository (see supplementary information).

Artefacts were categorised as blades when their length was twice their width, and dorsal flake scar ridges were oriented in a disto-proximal direction (after Shea, 2013: 32). Blades were classified as bladelets when the maximum width of the artefact was < 12 mm. Lithic remains with a maximum dimension of < 20 mm were classified as small flaking debris (SFD) and were counted and weighted. Technological length was only recorded when the artefact was transversally complete, whilst technological width was only recorded when the artefact was longitudinally complete.

Additionally, use-wear analysis was conducted on three points from Ha Makotoko’s Phase 9. They were excavated with use-trace analysis in mind and placed in airtight plastic bags immediately after removal from the soil. They were not touched, washed, or marked following their excavation. The tools were examined using an Olympus BX51M reflected light microscope with Nomarski prism, light polarising filter, bright- and dark-field application, and fitted with an Olympus SC30 digital camera. To record the various use-traces, magnifications of ×50, ×100, ×200, ×500, and ×1000 were used at different light exposures and polarisation/cross-polarisation filter positions. Micro-wear was recorded using the criteria described in Lombard (2005b) and the origin of micro-residues was investigated using the morphological characteristics published in Lombard (2005b, 2008).

Traces are interpreted as use-related only when multiple, supportive, and concomitant lines of evidence for their origins are observed (Lombard, 2011). Similar to other archaeological material, traces are interpreted as use-related within the context of the tool itself, i.e. layering on the tool, how they adhere to the tool surface/edge (i.e. were they originally wet, viscous, or dry), and where they are located in relation to tool morphology and other traces (e.g.Langejans, 2011; Wadley & Lombard, 2007). This multi-stranded, contextual approach also helps to eliminate ‘white noise’ or coincidental residues, fungal growth, and dust. Whereas micro-residues help identify contact or processed materials, striations or striated polishes are considered the most dependable indicator of use-direction or hafting configuration (Odell & Odell-Vereecken, 1980; Rots et al., 2006), and microfractures may indicate impact or hunting use (Lombard, 2005a).

#### Raw Material

Three different raw materials showed pronounced prevalence at Ha Makotoko: cryptocrystalline silicas (CCS, also referred to as opalines or chert, see, for example, Mitchell, 1996: 39), quartzite, and hornfels. Previous studies at Ha Makotoko indicated a dominance of CCS, and CCS indeed makes up 58% of all artefacts > 20 mm in Phases 9 and 10. This is, however, a much lower frequency than that recorded for the Later Stone Age assemblages from the Holocene layers at Ha Makotoko, where CCS makes up 80% or more in all phases (Mitchell & Arthur, 2014: 217). Hornfels and quartzite occur at a rate of about 21% each, with other raw materials occurring very rarely, mostly in the form of quartz.

However, these percentages look very different when considering the small flaking debris from the site: here, CCS dominates, with 4880 pieces (85.5%) weighing 948.4 g compared to 485 hornfels pieces (8.4%) weighing 134.5 g and 409 quartzite pieces (7.1%) weighing 139.3 g. These numbers indicate that at Ha Makotoko stone tool production was potentially focused on CCS as a raw material, with the lower quantities of hornfels and quartzite suggesting that some of the artefacts in these materials were produced elsewhere. This is despite the tendency of coarser grained materials (such as quartz) to fracture during detachment (Tallavaara et al., 2010; Will, 2021), potentially leading to more SFD when knapping with them.

This is further supported by the percentages of cortex prevalence. Of all artefacts with a maximum dimension of > 20 mm, 9.7% show cortex remains to some degree, 61% of which are CCS, 21.6% hornfels, and 17.4% quartzite. These percentages are similar to the raw material distribution across artefacts > 20 mm and are not as dominated by CCS as the small flaking debris (permutation‐based *χ*^2^ returns *p* = 0.89). Artefacts with > 33% cortex on the dorsal surface (or overall, on cores) are sparse, with only nine pieces made from CCS, six from hornfels, and one from quartzite identified.

It has previously been suggested that the CCS from nearby Rose Cottage Cave was primarily sourced from the river gravels of the broader Caledon River system of which the Phuthiatsana forms a key tributary (Clark, 1997). With 50% of the CCS cores from Ha Makotoko showing cortex remains, this proposition could also be considered for Ha Makotoko. However, the Ha Makotoko cortex types are not uniform, with both weathered and natural varieties of cortex present. It is striking that no pebble cortex can be found on the CCS cores from Ha Makotoko, indicating no direct exploitation of riverine raw material sources in the area. Around the site, on the other hand, a number of primary raw materials have been found, indicating that the exploitation of primarily terrestrial raw material sources is likely (Mitchell & Arthur, 2014).

#### Cores

**Fig. 4:**
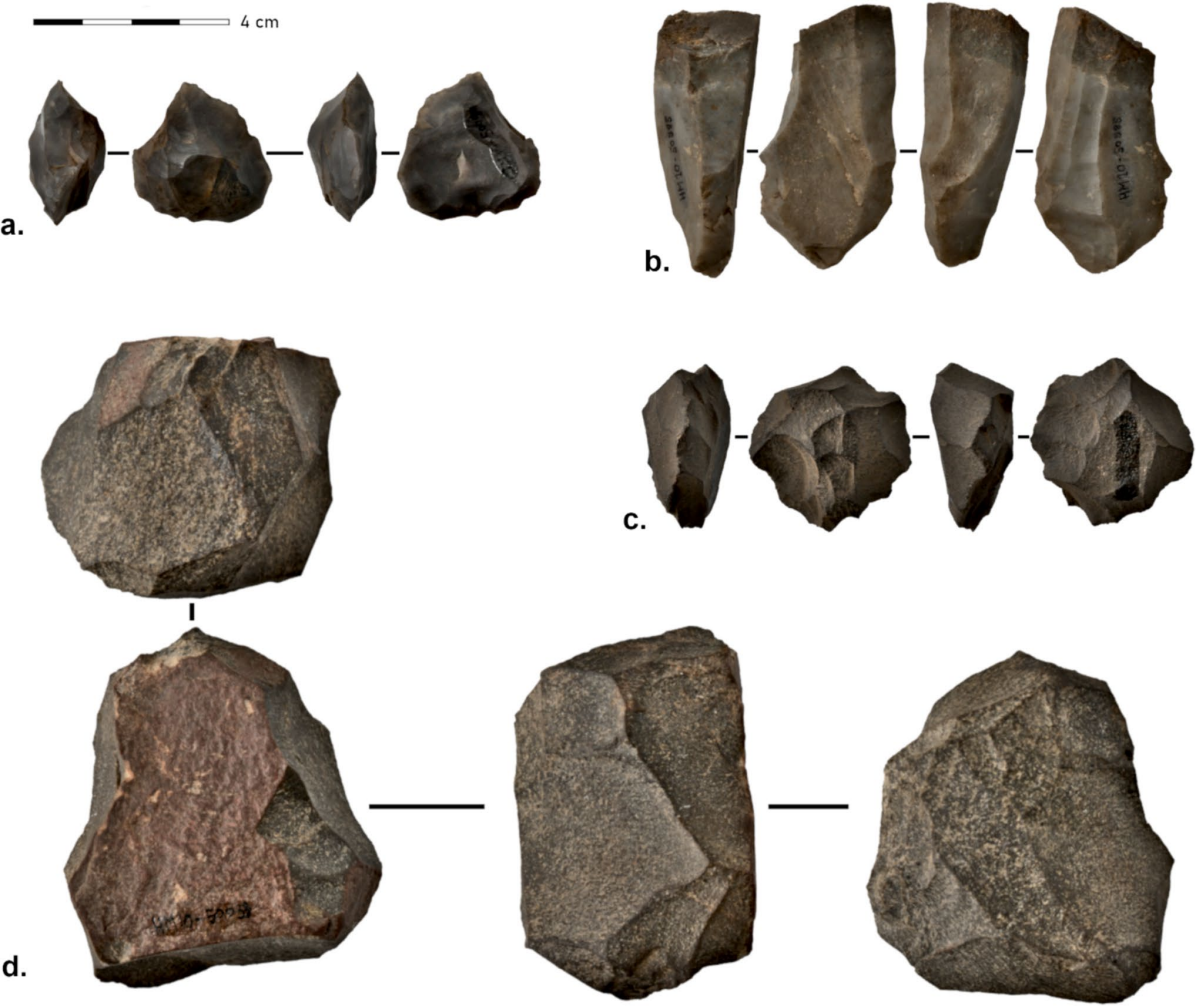
Cores from Ha Makotoko Phases 9 and 10: **a** Levallois core HM10-50087 (CCS). **b** Platform blade core HM10-50985 (CCS). **c** Discoid irregular core HM10-50772 (Hornfels). **d** Multipolar irregular core HM10-50033 (quartzite). 3D models of cores can be accessed via 10.5281/zenodo.17018398. Photographs: Siemssen

Overall, 27 cores were found in the MSA layers at Ha Makotoko (Fig. 4). Fourteen of these were quartzite, ten CCS, and three hornfels (Table 2). Thus, the over-representation of CCS found for SFD is not confirmed here. Collapsing hornfels and quartzite into coarse-grained material showed a strong mismatch between cores and SFD raw material (CCS = 10, coarse = 17 versus CCS = 4880, coarse-grained = 894). A chi-square test with 10,000 permutations testing between this distribution returned a highly significant result (*χ*^2^ = 45.8, *p* < 0.0001; Fisher’s exact *p* = 3.3 × 10⁻⁸, OR = 0.11), indicating a significant difference between the distribution of raw materials between cores and SFD.

**Table 2:** Ha Makotoko: core types, complete and proximal blanks, and tool types ordered by phase and raw material

**Phase 9 core type**	**CCS**	**Hornfels**	**Quartzite**	**Quartz**	**Other**	**Total**
Core-reduced piece	0	0	3	0	0	3
Irregular core	5	1	3	0	0	9
Levallois core	2	0	1	0	0	3
Platform blade core	0	0	0	0	0	0
**Total**	7	1	7	0	0	15
**Phase 10 core type**	**CCS**	**Hornfels**	**Quartzite**	**Quartz**	**Other**	**Total**
Core-reduced piece	2	0	0	0	0	2
Irregular core	0	0	1	0	0	1
Levallois core	1	1	6	0	0	8
Platform blade core	0	1	0	0	0	1
**Total**	3	2	7	0	0	12
**Phase 9 tool type**	**CCS**	**Hornfels**	**Quartzite**	**Quartz**	**Other**	**Total**
Point	1	1	0	0	0	2
Knife	0	0	2	0	0	2
MRP	3	4	8	0	0	15
Endscraper	0	0	1	0	0	1
Sidescraper	3	0	1	1	0	5
**Total**	7	5	12	1	0	25
**Phase 10 tool type**	**CCS**	**Hornfels**	**Quartzite**	**Quartz**	**Other**	**Total**
Point	1	0	0	0	0	1
Knife	1	0	0	0	0	1
MRP	6	2	1	0	0	9
Endscraper	0	0	0	0	0	0
Sidescraper	0	1	2	0	0	3
**Total**	8	3	3	0	0	14
**Phase 9 blank type**	**CCS**	**Hornfels**	**Quartzite**	**Quartz**	**Other**	**Total**
Blade	11	11	5	0	0	27
Bladelet	11	1	0	0	0	12
Flake	145	70	60	2	1	268
**Total**	167	82	55	2	1	307
**Phase 10 blank type**	**CCS**	**Hornfels**	**Quartzite**	**Quartz**	**Other**	**Total**
Blade	11	12	3	0	0	26
Bladelet	1	0	1	0	0	2
Flake	209	74	83	0	0	366
**Total**	221	86	87	0	0	394

Most of the cores made from quartzite are irregular and lack evidence of a clear organisation aimed at non-systematic, endstruck flake removal, with only one preferential Levallois core, one core-on-flake, and three (bipolar) core-reduced pieces present. Hornfels cores were made using both preferential Levallois and irregular techniques, whereas platform blade cores were only made in CCS. Preferential Levallois and irregular core types occur in both Phases 9 and 10, whereas platform blade cores, cores-on-flakes, and core-reduced pieces are confined to Phase 10.

**Fig. 5:**
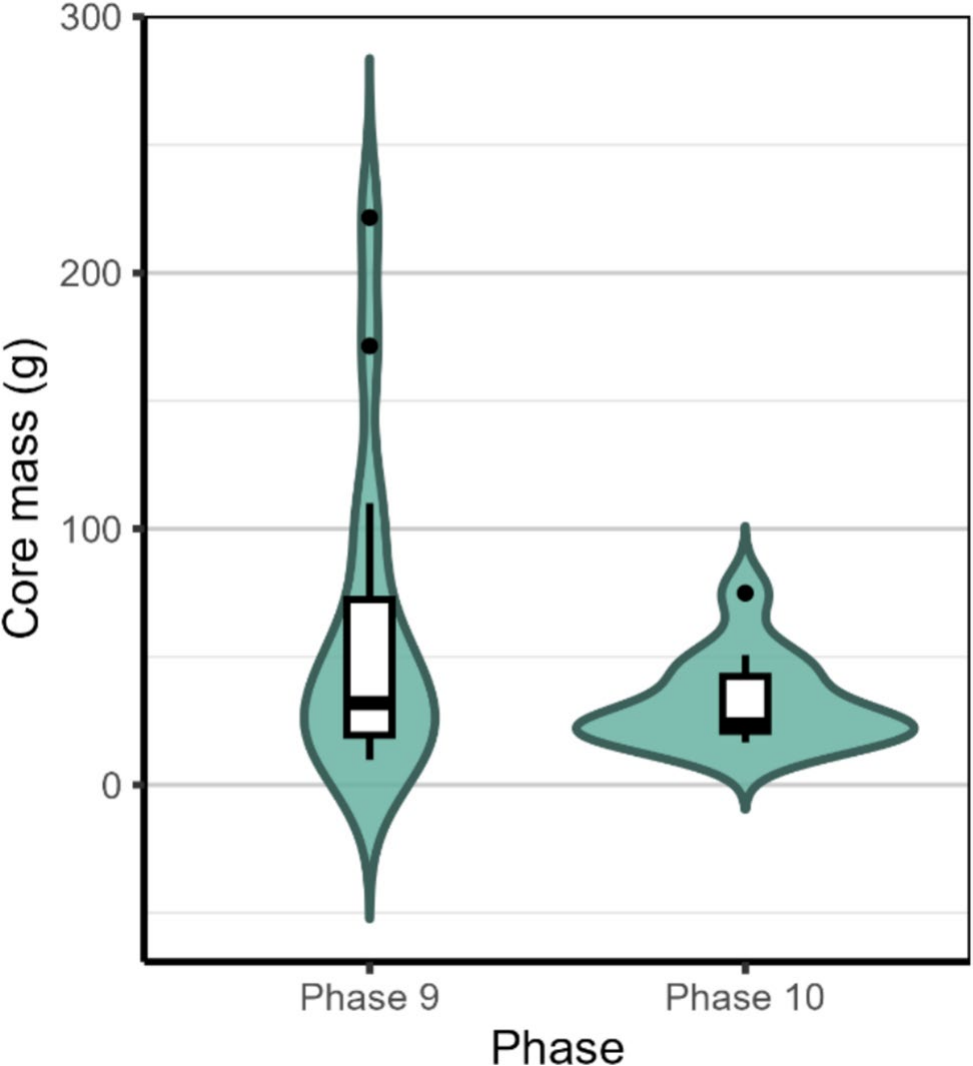
Left: Mass in gram of cores of Phases 9 and 10 at Ha Makotoko, showing higher median weight in Phase 9, than Phase 10

As the sample size is small, it is challenging to draw conclusions regarding differences in shaping technique throughout the two phases that are our focus here. This is particularly evident when discussing the prevalence of blades, which we address below. However, differences do emerge when looking at the weights of cores (Fig. 5). Here, Phase 10 shows smaller cores (Phase 10 mean = 32.3 g, median = 23.6 g vs. Phase 9 mean = 61 g, median = 31.9 g), despite a similar number of cores overall. However, a permutation-based *t* test confirms no significant mass differences between Phases 9 and 10 when discriminating between CCS and quartzite cores (CSS *p* value = 0.724; quartzite *p* value = 0.27), whilst there are indeed significant shifts in raw material use (Fisher’s exact test with Monte-Carlo simulation, *p* < 0.001), suggesting that the increase in core mass was caused by changing raw material configurations in Phase 9.

#### Blanks

**Fig. 6:**
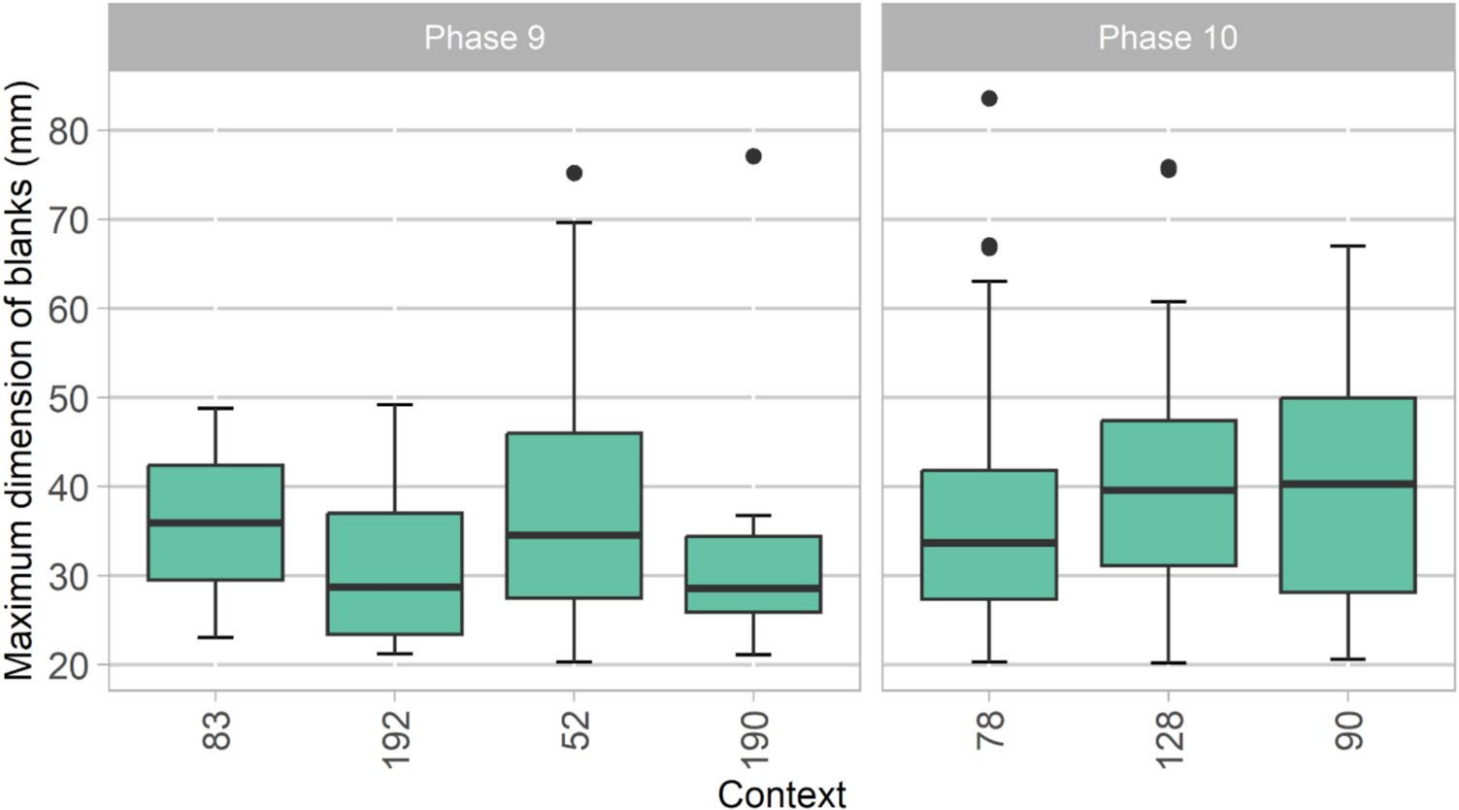
Maximum dimension of all complete flakes from the post-Howiesons Poort layers of Ha Makotoko, ordered by Context and Phase, showing some variation between Contexts but no vast changes overall

As the cores already indicate, the major objective of blank production in the post-Howiesons Poort layers of Ha Makotoko was flake production. Flakes overwhelmingly dominate these assemblages at Ha Makotoko, making up more than 89% of all proximal and complete blanks present (*n* = 634), although a low number of blades and bladelets (*n* = 53 and n = 14 respectively) are also present. Overall variability of the maximum dimension of complete flakes seems coherent throughout the stratigraphy, with similar variation occurring throughout all the contexts in Phases 9 and 10 (Fig. 6). Independent *t* tests show that maximum blank dimensions do not differ significantly between phases for CCS (*p* = 0.13), quartzite (*p* = 0.11), or hornfels (*p* = 0.54). Table 2 shows that bladelets are uncommon in the older Phase 10 but gain in relevance in the younger Phase 9.

Blanks at Ha Makotoko primarily show pronounced bulbs (73.8%) and few platform lips (8.2%), indicating the use of direct percussion by hard hammer, though such breakage patterns might vary significantly depending on raw material rather than knapping technique (Hahn, 1991; Moos et al., 2025). This is consistent with the expectations for this period of the MSA, when soft hammers seem to have been uncommon (Villa et al., 2005), and marks a distinct difference compared to the preceding Howiesons Poort complex, where soft hammer percussion was likely used for bifacial reduction in quartz Howiesons Poort points and blades (Soriano et al., 2007; de la Peña et al., 2013). Furthermore, 10.3% of all the complete flakes are convergent, a type of blank that has elsewhere been compared to the tool type of points (Timbrell et al., 2022, see also Will & Rathmann, 2025), a common characteristic of the post-Howiesons Poort.

#### Tools

Tools make up 2.7% of all the flakes present in Phases 9 and 10 at Ha Makotoko (*n* = 39). Fourteen of these are made on quartzite, 15 on CCS, nine on hornfels, and one on quartz. The tool types include sidescrapers, unifacially retouched points, an endscraper, miscellaneous retouched pieces (MRP), and knives (Table 2). Retouch is generally marginal. Whilst unifacially retouched points are, along with sidescrapers, among the most common tool types in post-Howiesons Poort assemblages (Porraz et al., 2013; Will et al., 2014), only three are present in Phases 9 and 10 at Ha Makotoko.

**Fig. 7:**
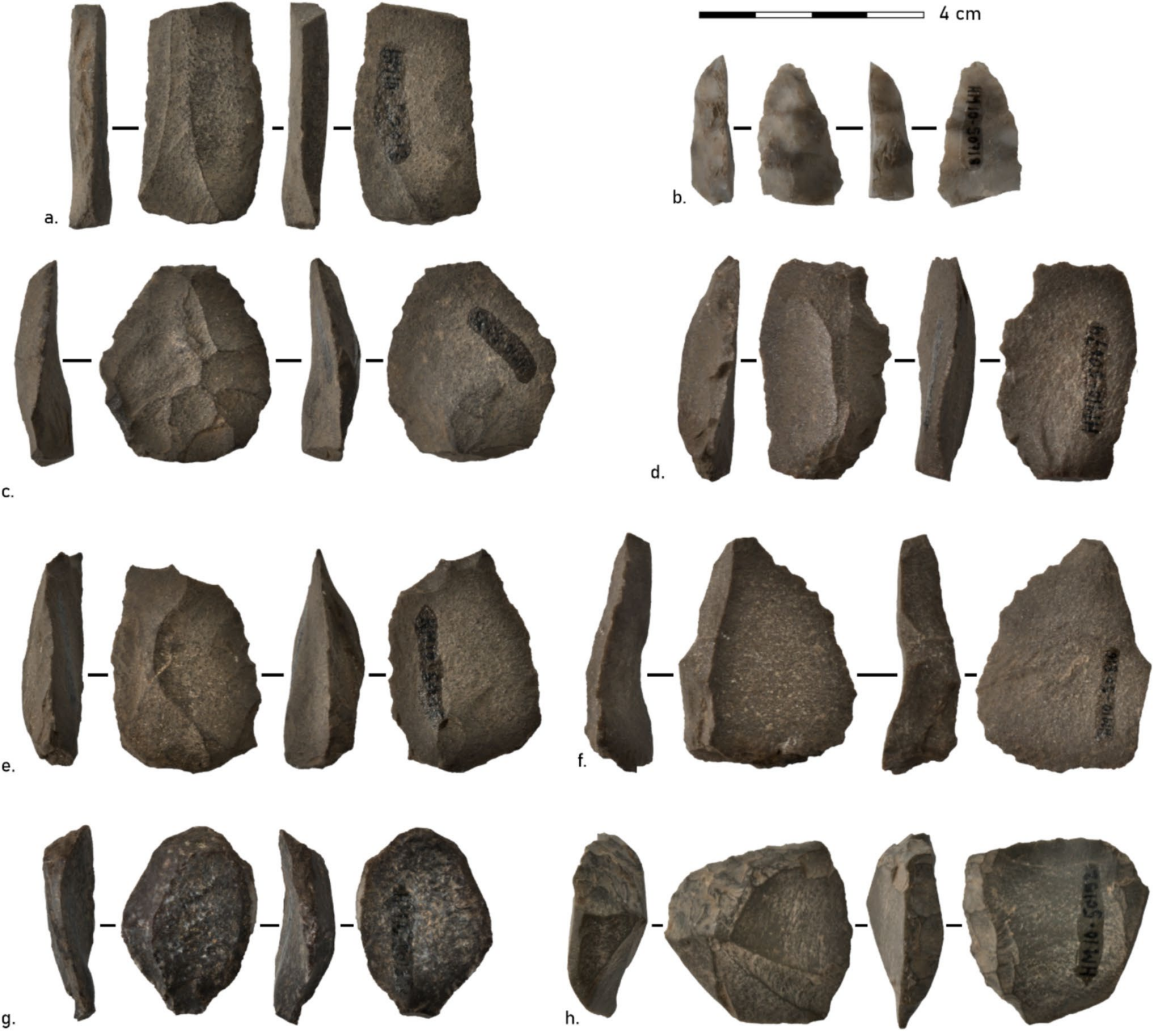
Tools from Phases 9 and 10 at Ha Makotoko: **a** Knife (HM10-50012; quartzite). **b** Point (HM10-50718; CCS). **c** MRP (HM10-50375; quartzite). **d** MRP (HM10-50674; quartzite). **e** MRP (HM10-50115; quartzite). **f** Sidescraper (HM10-50816; quartzite). **g** Sidescraper (HM10-50138; CCS). **h** Sidescraper (HM10-50192; CCS). Photographs: Siemssen

Since the initial postulation of the Sibudan technocomplex based on the post-Howiesons Poort material from Sibhudu Cave, KwaZulu-Natal, by Conard et al. (2012), four tool types allow for a positive diagnostic of this technocomplex: the Ndwedwe and the Tongati, as well as Naturally Backed Tools and Asymmetric Convergent Tools. However, none of the tools at Ha Makotoko shows strict similarities with these tool categories (Fig. 7), and with only four points available from Ha Makotoko, the presence of a Sibudan technocomplex cannot be postulated there. Instead, the high number of MRPs is particularly noteworthy for Ha Makotoko. Whilst no use-wear analysis was made on any of them, wooden fibre traces on points indicate that woodworking may have taken place at the site, for which the MRP may have been used.

#### Use-Wear

**Fig. 8:**
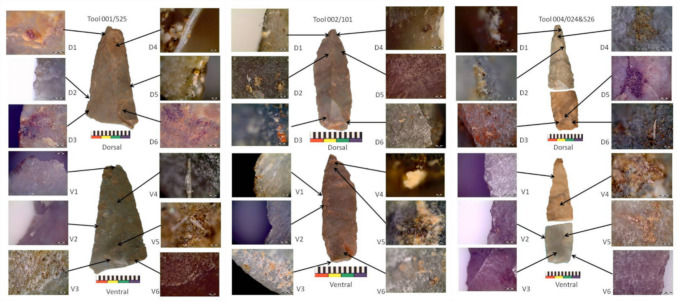
Points 001 (left), 002 (centre), and 004 (right) from Ha Makotoko Context 65 (Phase 9). Photographs: Lombard

Use-wear analysis was conducted on three of the points from Ha Makotoko (Fig. 8). Micrograph D2 of Point 001 shows edge damage caused by binding the point to a shaft. D5 shows a fatty animal residue in binding damage on the opposite side of the tool, which is supported by the fatty collagen on the ventral side illustrated in micrograph V2. Micrographs D3 and D6 show resinous deposits on the proximal portion of the tool, and V3 and V6 show bright polish, usually associated with friction against rigid, silica-rich plant material. Support for a wooden shaft is illustrated by the woody residue with intact silica skeleton shown in V5. The tool was probably used to process animal material. This is evident from the animal tissue recorded in micrograph D1, animal hair shown in D4, and the collagen fibre in V4. Although the tip shows some damage (V1), it is not clear whether this tool was primarily used for cutting or hunting activities, or perhaps both.

On Point 002, hafting is indicated by glue deposits situated on the proximal portion of the tool, indicated in micrographs D3, V3, and V6. Several glue deposits are associated with polished ridges as in D6, which might indicate a wooden shaft. Small red ochre particles are recorded with the glue deposits, see for example D3, but are too scarce to be certain of the use of a compound adhesive that included ochre as an aggregate (e.g. Lombard 2007). The processing of animal products is indicated by the preservation of fatty animal residues on the distal surface of the tool, illustrated in D2, V4, and V5, and along the distal edges associated with use damage, as shown in D4. Heavy-duty butchery may be indicated by the presence of collagen and/or bone residues near the tip, illustrated in D1, as well as by the diagonal striations in opposing (criss-cross) directions shown in D5. Such diagonal striations are often associated with sawing or heavy-duty cutting motions.

Point 004 is a refitted artefact that was probably broken as a result of a post-depositional event. Irregular damage and polish on the distal, ventral surface of the tool (V1) would seem to support an interpretation of post-depositional damage, such as trampling. This tool shows bright polish on the proximal ridges (V3), plus plant tissue and woody fibres associated with glue deposits on its surface (D5 and D6), as well as woody fibres along the proximal edges (V6), indicating that it was hafted to a wooden shaft. Some of the damage located along the proximal edges is associated with fatty animal residue (V2), which might indicate a sinew binding. The adhesive on this artefact contained red ochre (D3). Like the other points, it was probably used to process animal products as animal residues, including fat (D1), animal tissue and collagen (D2 and V4), and possibly blood (D4), have been recorded on its distal portion. It is uncertain whether this point was hafted as a butchering knife or as the tip of a hunting weapon.

#### Faunal Remains

Faunal assemblages from the MSA of southern Africa are commonly used to interpret palaeoenvironments (Faith, 2011), hunting behaviour (Clark & Kandel, 013; Dewar et al., 2024), and occupational changes on sites (Reynard, 2022). At Ha Makotoko, the post-Howiesons Poort layers yielded a range of faunal remains that are indicative of past ecological conditions. Identification took place to the lowest possible taxon by comparison with the African Mammal collection at the Royal Ontario Museum and the Savage Zooarchaeology Laboratory at the University of Toronto, Canada. The bone is highly fragmentary and primarily consists of unidentifiable pieces of trabecular bone and shaft fragments lacking evidence for a medullary cavity or diagnostic muscle markings. All identified mammal specimens were minimally assigned to Brain’s (1981) size classes: Size 1 = 5–23 kg, Size 2 = 23–84 kg, Size 3 = 84–296 kg, Size 4 > 296 kg; whilst small mammals are < 4.5 kg. In some cases, long bone fragments were attributed to size class based on cortical thickness (Reynard et al., 2014). To be recorded, long bone fragments had to present both an intact medullary cavity and periosteal cortical bone. The element, side, end, and proportion were recorded following Klein and Cruz-Uribe (1984) to calculate the number of identified specimens (NISP) and minimum number of individuals (MNI). Data were also collected following Marean et al. (2001) using the long bone zones published by Abe et al. (2002) in order to evaluate MNE (minimum number of elements) and MAU (minimum animal units). All evidence for taphonomic activity was recorded. Heat alteration was identified on the basis of colour changes such as yellow-red to purple-red for scorched bone that has been exposed to fires ranging from 300 to 550 °C, blue-black for charred bone exposed to 600 to 900 °C, and white for calcined bone exposed to temperatures > 1000 °C (Johnson, 1989; Shipman et al., 1984). Recording of carnivore activity, percussion marks, and cut marks followed Blumenschine et al. (1996) whilst that of weathering followed Behrensmeyer (1978). Long bone fragmentation angle and shape were also recorded (Villa & Mahieu, 1991) as well as evidence for root and acid etching.

The assemblage from Area 54 is dominated by bovids and includes both small and larger bovid remains (although Size 2 mammals dominate, weighing between 23 and 84 kg), small herbivores, birds, and a few remains of fish, suids, and equids (see Table 3). As many as 33.9% of the bones from the MSA layers at Ha Makotoko (*n* = 26,362) show signs of heat alteration, and 69.9% (*n* = 316) of long bones show green breaks, suggesting processing by humans, potentially related to marrow extraction (cf. Villa & Mahieu, 1991). Whilst the post-Howiesons Poort layers at Ha Makotoko yield a diverse range of species compared to the following sub-periods of the LSA, the time span covered is potentially much longer, and perhaps therefore also likely to encompass a wider variety of climatic and ecological conditions. The species found in Ha Makotoko’s MSA layers include plains zebra (*Equus quagga*), southern mountain reedbuck (*Redunca fulvorufula*), eland (*Taurotragus oryx*), red hartebeest (*Alcelaphus buselaphus*), bushbuck (*Tragelaphus sylvaticus*), grey duiker (*Sylvicapra grimmia*), klipspringer (*Oreotragus oreotragus*), steenbok (*Raphicerus campestris*), and warthog (*Phacochoerus africanus*). Riverine species (fish and frog) are present but much scarcer than during the later Holocene occupation of the site.

**Table 3:** Faunal remains of species collected from MSA Phases 9 and 10 of the 2010 excavation area at Ha Makotoko. NISP is Number of Identified Specimens and MNI is Minimum Number of Individuals

Area 54		Phase 9		Phase 10	
Taxon		NISP	MNI	NISP	MNI
Jackal	*Canis mesomelas*	1	1		
Suricate	*Suricata suricatta*			1	1
Size 2 carnivore		1	1		
Rock hyrax	*Procavia capensis*	2	2	1	1
Hare	*Lepus capensis*			2	1
Warthog	*Phacochoerus africanus*	1	1		
Suidae		1	1		
Equid sp.		2	1		
Red hartebeest	*Alcelaphus buselaphus*	1	1		
Alcelaphini		2	/		
Southern mountain reedbuck	*Redunca fulvorufula*	1	1		
Grey duiker	*Sylvicapra grimmia*	1	1		
Klipspringer	*Oreotragus oreotragus*	1	1		
Steenbok	*Raphicerus campestris*	1	1		
Bovid size 1		3	/	1	1
Bovid size 2		70	2	17	2
Bovid size 3		25	/	1	1
Ungulate size 2		2	/		
Ungulate size 3		1	/		
Small mammal		2	/		
Mammal size class 1		61	/	32	/
Mammal size class 2		210	/	149	1
Mammal size class 3		53	1	27	1
Mammal size class 4		3	1	4	1
Mammal		14,635		10,014	
Small fish		1	1		
Small bird	Song bird	2	1	1	1
Medium bird	Goose-sized	3	1	1	1
Large bird	Eagle owl size	2	1		
Small snake		6	1	1	1
Medium snake		5	1		
Tortoise		1	1		
Frog		6	1	2	1

Taken together, the macrofaunal remains from the post-Howiesons Poort layers of Ha Makotoko suggest an open dry grass and shrubland environment (cf. Skinner & Chimimba, 2005). This is further supported by the composition of the site’s microfaunal assemblage, which indicates temperate grassland, shrubs, and traces of a swampy environment. Microfaunal traces consist of vlei rat (*Otomys irrorattus*), forest shrew (*Myosorex varius*), and Namaqua rock rat (*Micaelamys namaquaensis*).

Lastly, carnivores also show some presence in the faunal assemblage. However, very few faunal remains show traces of carnivore bite marks, so it is plausible that carnivores like black-backed jackal (*Canis mesomelas*) were hunted and used for their fur. Whilst the small sample size prevents more robust analyses, the presence of small mammals (< 4.5 kg) and small territorial bovids suggests the use of snares (cf. Wadley, 2010), which could also account for the carnivores.

## Other Sites in the Area

With its location along the Phuthiatsana River, which drains from the Maloti-Drakensberg Mountains into the Caledon Valley, today Lesotho’s western boundary with South Africa, Ha Makokoto is located right at the interface of two contrasting biomes. These are the Afroalpine Maloti-Drakensberg Mountains to the east of the site and the open grasslands of the highveld further to its west. Contacts between sites within the Maloti-Drakensberg region such as Rose Cottage Cave and others along the South African coast to the east have been postulated for MIS 4 based on technological similarities (Way et al., 2022), but connectivity during MIS 3 is still subject to debate.

Here, we examine assemblages on multiple scales of analysis. First, the sites of Ntloana Tšoana and Rose Cottage are discussed, and the lithic assemblages from them are compared to the Phase 9/10 assemblage from Ha Makotoko. Second, the wider Maloti-Drakensberg Mountains are briefly reviewed, bearing in mind that only limited published evidence is currently available for material dating to MIS 3 from this region. Lastly, material from sites with more extensive MIS 3 records located nearer the South African coast in KwaZulu-Natal is examined, with the sites of Sibhudu, Umbeli Belli, Holley Shelter, Sibebe, and Border Cave discussed at the end of this section.

### The Caledon Valley

The Caledon River is located in the pre-Clarens Karoo basin and marks, as noted earlier, the border between the Free State province of South Africa and the western part of Lesotho (Bordy & Head, 2018). The Phuthiatsana drains into the Caledon from the east. Along with Ha Makotoko, a number of other MSA-yielding sites are known from the Caledon Basin, the most important of which are Ntloana Tšoana and Rose Cottage Cave. Both allow comparisons to be made to Ha Makotoko, given their close geographical and chronological relationships.

#### Ntloana Tšoana

Ntloana Tšoana was about 2 km east of Ha Makotoko along the Phuthiatsana River (Mitchell & Arthur, 2014). The site, now also drowned by the impoundment of the Metolong reservoir, was excavated by Mitchell and Steinberg in 1989 and later by Arthur in 2009–2012, although with a focus on the overlying LSA deposits (Arthur, 2018, 2022; Mitchell & Arthur, 2014; Mitchell & Steinberg, 1992). The HBL layer at Ntloana Tšoana precedes the MSA layers at Ha Makotoko, with a published OSL age of 56 ± 1.8 ka (Jacobs et al., 2008: S58) and a second, hitherto unpublished date of 57.8 ± 3.2 ka (Jacobs, pers. comm). The site yielded 2213 lithic artefacts (excluding small flaking debris of < 20 mm) associated with the MSA, and Mitchell and Steinberg (1992) note a great variability of retouch types contrasting with a low variability of raw material types.

Raw material proportions at Ntloana Tšoana differ significantly from those recorded at Ha Makotoko, with tuffaceous rock (recorded as quartzite in the 2010 Ha Makotoko excavation) being predominant at 50.2%. At Ntloana Tšoana, CCS occurred at a rate of 38.2%, with negligible percentages of hornfels, quartzite, and quartz in the post-Howiesons Poort layers (each > 1%). Cores are more similar to those from Rose Cottage Cave (see below), with Levallois forms being uncommon, but high rates of irregular cores like at Ha Makotoko. Blade and bladelet cores occur in low numbers. The post-Howiesons Poort assemblage at Ntloana Tšoana sees flakes as the dominant banks at 96.1% of all blanks, with 0.3% blades and 0.8% bladelets. Despite the stark contrast in raw material use at this site merely two km away, tools are similar overall and comprise knives, points, some scrapers, and backed pieces (cf. Mitchell & Steinberg, 1992).

#### Rose Cottage Cave

Rose Cottage Cave is located in the wider Caledon Valley around 35 km west of Ha Makotoko inside South Africa’s Free State province. The site has seen a long history of excavation and, with its stratigraphy reaching from the pre-Howiesons Poort into the LSA, offers an important record for understanding the southern African MSA. The post-Howiesons Poort layers relevant to this study were excavated by Harper (1994, 1997) in 1989. They were dated using thermoluminescence dating by Valladas et al. (2005: 169) on burnt lithics from the layers associated with the pre-Howiesons Poort, the Howiesons Poort, and the post-Howiesons Poort. The post-Howiesons Poort at Rose Cottage Cave was later dated to around 35–60 ka using OSL and radiocarbon dating (Loftus et al., 2019; Pienaar et al., 2008; Valladas et al., 2005; Wadley, 1995). Previously, Soriano et al. (2007) have documented diachronic trends throughout the Howiesons Poort and post-Howiesons Poort sequence at Rose Cottage Cave. Further excavations at Rose Cottage Cave are ongoing and more data on the post-Howiesons Poort will likely be available soon, allowing for further comparisons.

For this study, a subsample containing 50% of the material excavated in the Harper excavation in 1989 was analysed in the Evolutionary Studies Institute, University of the Witwatersrand, Johannesburg. The layers available for analysis that date to the post-Howiesons Poort were, from oldest to youngest, ANN, LIN, CLI, PAN, JEN, MAD, and JER. The younger layers LYN and KAR represent, just like MAD and JER, potentially very brief pulses of occupation (Harper, 1997: 471), but the former two assemblages were not available for analysis as part of this study. Given the high number of artefacts from these layers, the analysis was preceded by taking a subsample of a random 50% of all complete blanks from the post-HP layers at Rose Cottage Cave. These blanks then underwent similar metric and attribute analysis to the material from Ha Makotoko. The variables recorded for complete blanks were artefact class, raw material, maximum dimension, platform width, platform length, maximum width, and technological thickness. Further, core raw material and mass were recorded for all available cores from Rose Cottage Cave to allow for a comparison with those from Ha Makotoko. As no SFD remained with the artefacts from Rose Cottage Cave, a comparison of SFD proportionality will not be part of the discussion.

Raw material is similar to Ha Makotoko, but CCS comprises 87.3% of all lithic artefacts in the present sample, and Soriano et al. (2007) reported a CCS frequency of between 83.3 and 95.1% in the post-Howiesons Poort layers used for their technological analysis. At Rose Cottage Cave, more flakes with cortex remains can be found than at Ha Makotoko, and cortex is present on 21.3% of all flakes in the sample used for this study.

**Fig. 9:**
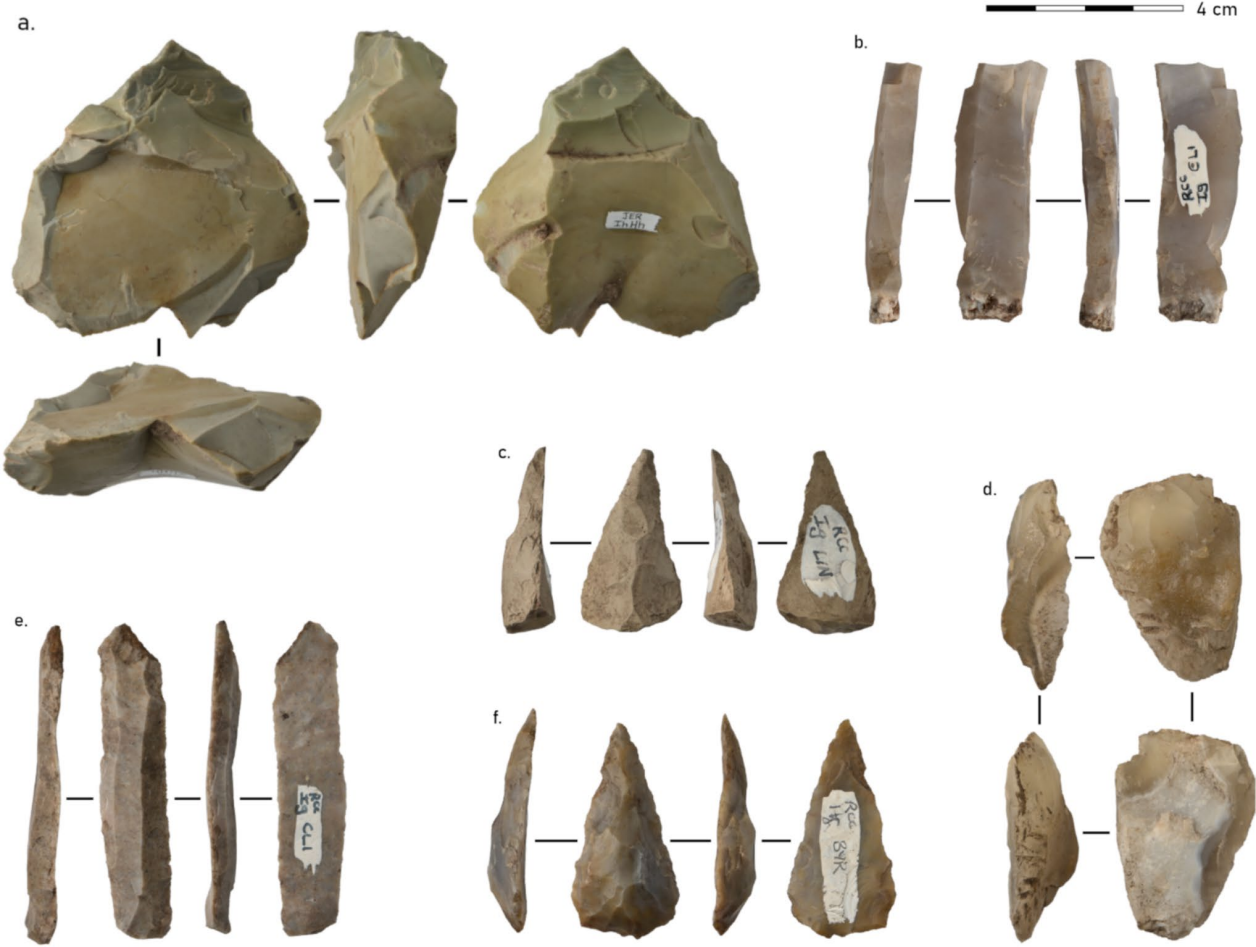
Cores and tools from Rose Cottage Cave: **a** Irregular/Levallois core RCC-HP94355. **b** Platform blade core RCC-HP94141. **c** Point (RCC-HP-LIN-001). **d** Core-reduced piece (RCC-HP-JER-002). **e** Retouched blade (RCC-HP-CLI-002). **f** Point (RCC-HP-BYR-001). 3D models of cores can be accessed via 10.5281/zenodo.17018398. Photographs: Siemssen

Cores from Rose Cottage Cave are, on average, much smaller and lighter than those at Ha Makotoko (Ha Makotoko core mass mean = 48.2 g versus Rose Cottage Cave core mass mean = 12.6 g). This tendency does not seem to vary chronologically but persists throughout the different post-Howiesons Poort layers at the site. A permutation-based *t* test cannot confirm a statistical difference of core weights between raw material classes at Rose Cottage Cave (*p* = 0.176). As at Ha Makotoko, Harper (1997) reports that core types at Rose Cottage Cave are dominated by irregular cores at 55.6% of all cores (*n* = 185), whereas platform blade cores are more frequent here at 32.1% (*n* = 107; Fig. 9). Soriano et al. (2007) document the use of a bipolar knapping technique starting with the post-Howiesons Poort of Rose Cottage Cave, and flakes and blades produced using it persist throughout the post-Howiesons Poort layers of the sequence.

**Fig. 10:**
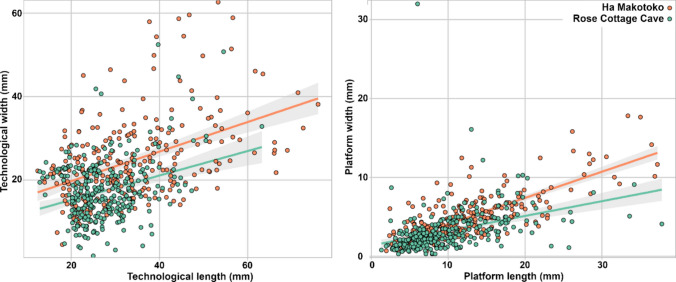
Technological length and maximum width and platform length and width of all complete blanks measured at Ha Makotoko and Rose Cottage Cave

Flakes are shorter and less wide at Rose Cottage Cave, and at the same time, platforms are also shorter and thinner (Fig. 10). This tendency seems to correlate with the smaller core size. The percentage of blades and bladelets is higher at Rose Cottage Cave at 4.3% and 7.1%, respectively. However, flake production seems to have been the major objective of stone tool production. A permutation-based linear-by-linear association test performed on all complete flakes and blanks ordered by level returns a p value of 0.06, suggesting no significant difference in the maximum dimension of complete blanks (including flakes, blades, and bladelets) over time at Rose Cottage Cave.

Many more tools are available from Rose Cottage Cave than from Ha Makotoko. Those from the site’s post-Howiesons Poort layers make up just under 4% of the assemblage (Harper, 997) and are dominated by scrapers, followed by knives, unifacially retouched points (with individual pieces showing marginal bifacial retouch), and miscellaneously retouched pieces (Fig. 9). All these types are also present in the MIS 3 layers of Ha Makotoko, but occur there in much smaller numbers. However, a lack or scarcity of formal tools is not unusual for the post-Howiesons Poort (Timbrell et al., 2022).

### The Wider Maloti-Drakensberg Region

Within the wider Maloti-Drakensberg region, several other MIS 3 sites are relevant to the discussion of Ha Makotoko. Most importantly, these include Sehonghong and Melikane (both located in eastern Lesotho), and Strathalan B (located in the Eastern Cape Province of South Africa). The oldest layers yet published in detail from Sehonghong (Layers RFS, MOS, OS, dated between 30 and 24 ka) are considered transitional between the LSA and MSA (Mitchell, 1994; Pargeter et al., 2017) and comprise lithic artefacts similar to the material found at Ha Makotoko, being mostly made of CCS, with lower percentages of hornfels, dolerite, quartz, and others. The older layer RFS features, along with a heavy use of CCS, a pronounced use of dolerite dyke material. The use of CCS decreases in MIS 2, and hornfels gains importance (Carter et al., 1988). Most of the cores are irregular, and the bipolar production technique was used throughout all three layers (Mitchell, 1994). Again, most blanks are flakes, with very few blades and bladelets found. The mean size of unmodified CCS blades and flakes increases slightly over time, with a mean size of 25.5 mm (blades) and 22.03 mm (flakes) found for the older layer RFS. No points have been recorded for layers RFS to OS at Sehonghong, but backed knives do occur (Mitchell, 1994). Carter et al. (1988) reported tools as comprising only 1% of the MSA assemblage but the analysis was limited in scope and the stratigraphic controls were poor, given that the original 1971 excavations proceeded in 10-cm-thick spits that cross-cut the site’s complex natural stratigraphy. More recently, much better-controlled excavations by Stewart and Dewar have reached back as far as 33 ka, but details remain unpublished and underlying deposits have yet to be investigated (Loftus et al., 2015).

The site of Melikane saw multiple occupational pulses throughout the MSA from MIS 5a into MIS 2. Initially excavated by Patrick Carter in 1974 (again in 10-cm-thick spits), it was re-investigated by Brian Stewart and Genevieve Dewar in 2007–2009 (Stewart et al., 2012, 2016; Pazan et al., 2022, 2023). However, little has been published thus far regarding the two occupational pulses corresponding to MIS 3, comprising layers 15–22 and 6–14, which are dated to c. 51 and c. 38 ka respectively (compare Pazan, 2022). Whilst overlying assemblages dating to the early part of the Last Glacial Maximum have received considerable attention at Melikane, the MIS 3 assemblages are still to be analysed in-depth (Pazan et al., 2022, 2023; Stewart et al., 2012). Pazan et al. (2023) nevertheless point out intra-site continuities relating to the preparation of platforms and the production of microliths. Despite stark technological contrasts between phases, the frequency of platform preparation broadly seems to decrease between MIS 5a and MIS 2, whereas miniaturisation of lithic artefacts seems to increase as early as MIS 5a. Whilst the onset of an Early Later Stone Age has been postulated to begin around 45 ka at Border Cave (d’Errico et al., 2012; Villa et al., 2012), Pazan et al. (2023) note that several of its key components (e.g. microlithisation, bipolar cores, and scaled pieces (*pièces esquillées*) gradually appear some 40,000 years earlier at Melikane, leading them to reject the idea of a strict technological boundary between MSA and LSA stoneworking technologies (Pazan et al., 2022, 2023; cf. Bader et al., 2024; Mitchell, 2024), something previously suggested by Clark (1997) for the early MIS 2 layers of Rose Cottage Cave, by Mitchell (1994) with regard to assemblages from Sehonghong, and by de la Peña (2015) with regard to the appearance of the bipolar knapping technique in MIS 3 as a whole.

Strathalan B is dated to the end of MIS 3, with its lower layer VBA dated to around 32 ka (Opperman, 1996). Most of its artefacts are made from hornfels, with a few made from chalcedony and other raw materials. Cores are irregular, and the assemblage is again dominated by flakes. Strathalan B offers good preservation of organic remains and is currently undergoing re-excavation (Reynard et al., 2024a).

### Southeastern Southern Africa

Nearer the southeastern coast of South Africa, in KwaZulu-Natal, three sites—Sibhudu, Umbeli Belli, and Border Cave—show extensive archaeological records broadly contemporary with the post-Howiesons assemblages from Ha Makotoko. Deposits at two other sites—Umhlatuzana and Holley Shelter—also fall into MIS 3 and could aid broader comparisons in future studies (Bader & Will, 2022; Bader et al., 2015; Kaplan, 1990; Sifogeorgaki et al., 2020).

#### Sibhudu

Sibhudu is located around 40 km north of Durban along the Tongati River (Wadley, 2005). With 23 horizons associated with the post-Howiesons Poort, Sibhudu marks one of the most extensive available post-Howiesons Poort sequences known, and various aspects of their stone artefacts have found attention in the literature (Wadley, 2005; Wadley & Jacobs, 2006; Will & Conard, 2018). The post-Howiesons Poort alone comprises 23 layers (layers RB–BSP) that are dated from c. 58 to 57 ka (Will & Conard, 2018). This material was used to define the Sibudan technocomplex and has been subject to various modes of analysis. *Inter alia*, the hafting traces of Sibhudu points have been analysed by Lombard 2005a, and its bone tools have also found their way into discussion, in particular as compared to those from the Howiesons Poort layers below (de la Peña & Wadley, 2017).

The late MSA layer RSP from Sibhudu is dated to around 46 ka and follows the post-Howiesons Poort sequence after a hiatus of about 10,000 years (Jacobs et al., 2008). The lithic assemblage from this layer was published in detail by Villa et al. (2005). They note that dolerite and hornfels are the most common raw materials, which is congruent with the lower post-Howiesons Poort layers and, as at Rose Cottage Cave and Ha Makotoko, marks the exploitation of local raw material resources (see also Porraz et al., 2013). Cores include those that show recurrent unidirectional or bidirectional flaking, as well as bladelet cores, the latter exclusively comprising hornfels. Hard hammer percussion dominates the assemblage, but some soft hammer percussion has also been observed. A striking difference to the MSA at Ha Makotoko is the focus on blades, with laminar products forming around 37% of all blanks (Villa et al., 2005). Retouched tools primarily comprise points (32.8%, of which two pieces show some bifacial retouch), as well as lower percentages of sidescrapers (16.6%) and MRPs (16%). At 15%, the frequency of tools is high in the RSP layer (Villa et al., 2005). This is similar to the post-Howiesons Poort layers underneath, but marks a distinct difference to the low tool percentages found at Ha Makotoko.

#### Umbeli Belli

Umbeli Belli is located around 90 km south of Sibhudu and some 7 km from today’s shoreline and has an occupational history spanning from the MSA into the LSA. Excavations started in 1979 under the direction of Charles Cable (1984) and more recent excavations from 2016 to 2020 reached bedrock. Umbeli Belli’s lowest geological horizon GH10 is dated to c. 54-47 ka (Bader et al., 2018) and contains a late MSA assemblage (Tribolo et al., 2024; Bader et al., 2018, 2022b).

Cores were exploited until little remained, and with 6% of flakes showing more than 50% cortex, Bader et al. (2022b) expect the site to have been used for stone tool production. Raw material may partially have been sourced from the nearby Mpambanyoni River, which is indicated by the pebble cortex of the artefacts. Raw materials in GH10 are dominated by quartzite at 67.8%, followed by hornfels at 24.3% and quartz at 4.1%. Cores were primarily prepared with a single striking platform that is accompanied by a wide removal surface (Bader et al., 2022b), like at Sibhudu and also seen in at least one example (core HM10-50033) from Ha Makotoko (Fig. 4). At Umbeli Belli, flakes make up 88.1% of blanks, followed by blades at 8.8% and bladelets at 1.7%. Points were recorded separately and make up 1.4% of all the lithic blanks present. Retouched tools make up 3% of all the artefacts in GH10, but include very few bifacial tools (Bader et al., 2022b). Other formal tools present include scrapers and backed knives as well as a range of points (straight base unifacial points, narrow points, and a double tipped bifacial point).

Comparisons between Umbeli Belli and Sibhudu have evidenced (late) post-Howiesons Poort local variation, which fits well with the results of this comparison between Ha Makotoko and Rose Cottage Cave (Bader et al., 2022b). The technological change that occurs at Sibhudu was heterogeneous in character, with both abrupt and gradual change occurring (Conard & Will, 2015; Will & Conard, 2018).

#### Border Cave

Border Cave is located along the border between KwaZulu-Natal and eSwatini. Having been excavated since the 1930s, the site has seen renewed excavations since 2015 (Backwell et al., 2022). Noted for its exceptional preservation that has yielded a rich organic record—and, in particular, human remains—the stratigraphy at Border Cave encompasses layers reaching from the pre-Howiesons Poort into the final MSA, corresponding to MIS 7–2 (de la Peña et al., 2022; Zwane & Bamford, 2021). Border Cave’s Members 2 WA, 2 BS Lower A, B, and C, and 2 BS Upper date between 60 and 39 ka and correspond to the post-Howiesons Poort (Backwell et al., 2018). Whilst some scholars consider the younger of those layers (postdating 44 ka) as transitional between the MSA and the LSA (Villa et al., 2012), this assessment is not univocally shared (cf. Bader et al., 2022a; Mitchell, 2024).

The raw materials used in the post-Howiesons Poort assemblages at Border Cave are dominated by fine-grained rhyolite, with basalt and hornfels making up most of the remaining artefacts (de la Peña et al., 2022). Cores consist of Levallois, blade, and irregular forms. Blank production is again dominated by flakes, which make up around 61.4% of the assemblage, followed by blades with 31.9%. Blank production was exercised by direct percussion with a hard hammer, marking a distinct difference to the marginal percussion used previously during the Howiesons Poort (Villa et al., 2012). This is similar to the other sites evaluated for this study. In the oldest post-Howiesons Poort layers, unifacial points and pointed blades are common, along with scrapers and denticulates (Villa et al., 2012).

The post-Howiesons Poort layers above, ranging from around 49 to 45 ka, display a shift in the tool spectrum, with unifacial points disappearing in younger layers and lower numbers of scrapers and denticulates. In turn, bifacial knapping becomes more common and the raw material choices change towards an increased use of chalcedony. Discoid cores are more common here (Villa et al., 2012). Timbrell et al. (2022) note increased technological diversity as one of the key characteristics of the post-Howiesons Poort at Border Cave and elsewhere.

#### Sibebe

Sibebe, in eSwatini, features a late to final MSA sequence dated between 43 and 27 ka. Following extensive excavations in the 1960s by Beaumont, the site has recently been re-excavated (Bader et al., 2022b). The site shows a dominant use of quartz throughout the layers, and quartz makes up between 78 and 93% of the raw material proportion. The bipolar knapping technique is uncommon at first, but more common in the upper MSA and LSA section. At Sibebe, flakes occur most frequently, and the lowest MSA layers show this blank prominence most clearly. Blades gain prominence throughout the sequence, but their percentage remains below 20% throughout the MSA sequence. Tools range around 4% throughout the sequence but increase to 9% in the higher MSA levels. Especially towards the later MSA, unifacially and bifacially retouched points occur, whereas other retouched tools are infrequent (Bader et al., 2022b).

#### Holley Shelter

Excavations at Holley Shelter, located in KwaZulu-Natal, gave light to both MSA and LSA material. Initially excavated in the 1950s, the site recently saw re-excavations, and its two uppermost MSA layers AVA and BIB have been dated to between 34 and 36 ka (Bader et al., 2024), some 5 k years younger than the Phase 9 assemblage of Ha Makotoko. The assemblage at Holley Shelter is dominated by hornfels and tuned towards laminar production (where blades and bladelets make up > 30% of the AVA and BIB layer associated with Holley Shelter’s final MSA). This marks a distinct difference not only to the primarily flake-based industries found at Ha Makotoko, Rose Cottage Cave, and Sibhudu dated slightly older, but also stands in stark contrast to the later MSA expressions found at Border Cave, or Sibebe, that see a burgeoning of bifacially retouched points. Bipolar production occurs in both layers, with unifacial points and retouched blades the most common tools present (Bader et al., 2024).

## Discussion

Based on our chrono-chorological comparison, we argue that Ha Makotoko integrates well with known post-Howiesons Poort assemblages and shows some components of final MSA assemblages in the region (such as some backed tools). However, there are distinct differences, as well as potential specificities, with respect to Phases 9 and 10 at Ha Makotoko. Firstly, no bifacial points are known from Ha Makotoko’s Phase 9 and 10, despite its younger contexts being largely contemporaneous to occupations nearby or on the eastern coast (e.g. at Sibhudu, or Sibebe) that show bifacial point production shortly thereafter (cf. Bader et al., 2024). Instead, the assemblage seems more congruent with slightly older post-Howiesons Poort assemblages with a heavy focus on flake production, although no ‘Sibudan’ points have been recorded at Ha Makotoko.

Furthermore, Ha Makotoko shows some signs of the focused bladelet production associated with final MSA assemblages from further east at Melikane, Border Cave, or at Sibebe (Bader et al., 2024; Pazan et al., 2023; Villa et al., 2012), as bladelet production increases throughout its Phase 9. Most notably, though, Ha Makotoko shows a higher-than-usual frequency of miscellaneous retouch inconsistent with formal tool typologies, of which some show notches sometimes associated with woodworking industries. This potential focus on wooden artefacts is also reflected in use-wear traces found on points from the site that show polish associated with the use of wood.

Ha Makotoko’s Phases 9 and 10 thus appear to be a regionally specific expression of the post-Howiesons Poort to (early) final MSA, characterised by flake production, irregular cores, and miscellaneous retouch. Along with scrapers and unifacially retouched points, a backed knife completes this MIS 3 assemblage. Use-wear analysis confirmed that unifacially retouched points were hafted to wooden shafts, revealing a woodworking industry in the form of shaft making, although the points themselves were probably used for animal processing. Indeed, one might interpret this site as potentially being, more than elsewhere, focused on the production of non-lithic artefacts: the high frequency of MRPs with notches, the use-wear traces on points confirming wood use, and the occurrence of core-reduced flakes that have elsewhere been interpreted as ‘wedges’ for splitting wood (cf. Shea, 2013) could indicate that a far larger body of material than the surviving stone tools has been lost to preservation bias throughout the MIS 3 sequence of Ha Makotoko.

Discussion of the archaeology of MIS 3 has seen a contentious and somewhat condescending research history, with the post-Howiesons Poort previously described as a ‘devolution’ of lithic technology that reflects a cognitive cul-de-sac (Henshilwood, 2007: 130, but see Lombard & Parsons, 2010, 2011). Despite this, more recent studies agree that there was no ‘devolution’ of stone tool technology following the Howiesons Poort (de la Peña & Wadley, 2017; Kandel et al., 2016; Pazan, 2022; Will et al., 2014), as more light is being shed on the variability of technology throughout this period. Indeed, the change from the Howiesons Poort to the post-Howiesons Poort is no longer understood to be an all-encompassing technological transition. Instead, certain technological aspects remain visible in the archaeological record both during the transition from MIS 4 to MIS 3 and throughout MIS 3, and change appears to have been gradual, rather than sudden. At Ha Makotoko, this is visible in a continued use of unifacial points and continued focus on irregular core reduction from early to mid-MIS 3. At Rose Cottage Cave, raw material configuration remains broadly similar between MIS 4 and MIS 3, a phenomenon that can also be observed at sites such as Diepkloof and Klasies River Cave in South Africa’s Cape (Soriano et al., 2007; Lombard & Parsons, 2010; Porraz et al., 2013).

The technological changes evident in MIS 3 are commonly interpreted to have been driven by changing ecological conditions (Mackay, 2011). However, whilst the post-Howiesons Poort corresponds broadly to the first half of MIS 3, the onset times for this phenomenon vary widely across southern Africa. Whereas this is dated to around 57 ka in Lesotho (although dates scatter at Rose Cottage Cave, see Jacobs et al., 2008; Pienaar et al., 2008), the post-Howiesons Poort appears earlier along the coast of KwaZulu-Natal (Lombard et al., 2022). At Klasies River, the post-Howiesons Poort sees its onset before larger climatic shifts are visible in the microfauna record from the site (Reynard et al., 2024b).

Additionally, Stewart and Mitchell (2018) have illustrated how ecological conditions varied considerably during MIS 3 in the Maloti-Drakensberg region as a whole, something that further complicates investigations into potential correlations between technological and environmental changes. Whilst MIS 3 shows higher temperatures compared to the preceding MIS 4 on a global scale, this does not translate simply onto the local or regional level. In the Maloti-Drakensberg region, MIS 3 is particularly associated with an elevated variability in rain patterns and is understood to have been a climatically volatile period (Stewart & Mitchell, 2018). Here, vegetational conditions are closely coupled to temperature fluctuations in the mountainous environment of Lesotho, whereas other parts of southern Africa see precipitation as a main driver of ecological change (Patalano et al., 2023; cf. Clark 2013). MIS 3 thus presents a period of persistent ecological, technological, and socio-cognitive change. Whilst those changes appear differently on various scales, they are nonetheless inseparable from each other (Roberts, 2023).

De la Peña and Wadley (2017) associate the Howiesons Poort to post-Howiesons Poort transition with changing mobility patterns at Sibhudu due to the poor correlation there between technological and environmental records. Dusseldorp (2014), in turn, associates the post-Howiesons Poort with subsistence changes that thematically align with Sahle and Lombard’s (2024) postulation of a change in weapon-associated hunting ranges. Their work shows that backed microliths were best suited ballistically for maximum-range bowhunting and long-range javelin hunting, whereas post-Howiesons Poort points were better suited for medium- or short-range hunting spears or javelins. A partial abandonment of this technology in early MIS 3, visible in the disappearance of small segments at the onset of the post-Howiesons Poort (e.g. at Rose Cottage Cave) and the cessation of widespread use of backed retouch, may correspond to socio-economic shifts such as hunting larger game packages, as seen at Sibhudu (Clark & Plug, 2008). In addition, materials used for arrow production may have changed, and small bone points appear in greater numbers at MIS 3 sites such as Border Cave and White Painting Shelter (d’Errico et al., 2012; Robbins et al., 2012).

These changes further correlate with an increasing fragmentation of social structures noted by Mackay et al. (2014), who see a localisation of knowledge exchange evidenced by the archaeological record of MIS 3 (cf. Way et al., 2022), perhaps associated with genetically observed fluctuations in population sizes (Schlebusch et al., 2020). Additionally, the relevance of the post-Howiesons Poort for investigations into human cognition was noted almost two decades ago (Lombard & Parsons, 2010, 2011, cf. Henshilwood, 2007), but the scientific community has struggled to draw conclusions on the cognition of MIS 3 more recently despite some attempts to introduce a ‘cognitive archaeology of the MSA’ (cf. d’Errico & Colagè, 2018; Colagè & d’Errico, 2020; Wadley, 2021; Lombard & Gärdenförs, 2023; Roberts, 2023; Lombard, 2025). Whilst we do not aim to contribute to this debate with our study here, we emphasise that the findings from Ha Makotoko need to be regarded as embedded in wider environmental and socio-cognitive ecologies, rather than in isolation from them.

## Conclusion

The lithic assemblage from the western Lesotho site of Ha Makotoko described in this study offers additional evidence for a time period of the southern African Middle Stone Age that still yearns to be understood by archaeologists working across the sub-continent. With its increasing technological heterogeneity compared to the preceding Howiesons Poort, the MSA of MIS 3 defies the application of unidirectional evolutionary narratives to changing technological traditions.

At Ha Makotoko, the MSA assemblages from Phases 9 and 10 dating to c. 42 ka and > 52 ka are characterised by a prevalence of flakes, with few platform blade cores and blades found. The tools found in these layers comprise points, sidescrapers, and a notably high amount of informal retouch. CCS was widely used in addition to quartzite, and low percentages of hornfels and quartz are also present. Use-wear traces on the points from Phase 9 indicate hafting and a use in both butchery of animals and woodworking. The MIS 3 lithic assemblage was found along faunal remains indicating the presence of a grassland fauna. Statistically confirmed technological similarities indicate a close relationship between the two Phases despite their stratigraphic separation and the ambiguity of the older date, which approaches the limit of radiocarbon dating at > 52 ka. The sequence nevertheless also shows some change, with bladelets increasing in relevance throughout it.

Generally, Ha Makotoko integrates well with our current fractured understanding of the post-Howiesons Poort and final MSA elsewhere, but offers some anomalies, such as the high number of MRPs, or the lack of bifacial retouch that gains relevance throughout this period at other sites nearby. Despite this, direct comparisons to lithic assemblages in the Caledon Valley, the wider Maloti-Drakensberg Mountains, and sites along South Africa’s southeastern coast in KwaZulu-Natal show distinct similarities in both the tool spectrum present, for example with unifacial points occurring widely throughout the earlier part of MIS 3 and an increasing frequency of bladelets overall. Indeed, Ha Makotoko further complexifies a period that is already known for its variable technological record. Further research on regional chronologies, interaction, and the environmental, cognitive, and technological feedback loops responsible for these transitions and continuities is clearly necessary to do justice to the archaeology of MIS 3.

## Supplementary Information

Below is the link to the electronic supplementary material.

ESM 1 3D models of cores from Ha Makotoko and Rose Cottage Cave recorded for this study are available via https://doi.org/10.5281/zenodo.17018398. Additional supplementary data are available upon request. (CSV 270 KB)

## Data Availability

3D models of cores from Ha Makotoko and Rose Cottage Cave recorded for this study are available via 10.5281/zenodo.17018398. Additional supplementary data are available upon request.
